# Experience and Acceptance of Cosmetic Procedures Among South Korean Women in Their 20s

**DOI:** 10.1007/s00266-018-1257-0

**Published:** 2018-10-25

**Authors:** Yun Am Seo, Hyang-In Cho Chung, Young A Kim

**Affiliations:** 1 Applied Meteorology Research Division, National Institute of Meteorological Sciences, Seogwipo-si, Jeju-do Republic of Korea; 2 College of Nursing, Chonnam National University, Gwangju, Republic of Korea; 3 College of Nursing, Jeju National University, 102 Jejudaehakno, Jeju-si, Jeju-do 63243 Republic of Korea

**Keywords:** Acceptance, Attitude, Aesthetic, Cosmetic, Plastic surgery, Women

## Abstract

**Purpose:**

This study aimed to investigate the relationship between acceptance of cosmetic surgery (ACS) and cosmetic procedure experience in women in their 20s.

**Methods:**

We collected 330 randomly sampled women in their 20s in November 2017. The collected data were analyzed by descriptive statistics, *t* test, crosstabs, Chi-square test, ANOVA, Pearson’s correlation, and binary logistic regression.

**Results:**

Almost all (97.9%) of the subjects had heard about the side effects of cosmetic surgery, and “Internet” was the most common source of information (57.3%). The number of subjects who had already undergone more than 1 cosmetic procedure was 124 women (37.6%), and the first age of cosmetic procedure was 21.81 (± 2.83) years old. ACS had a negative correlation with appearance satisfaction (*r* = − 0.18, *p *= 0.001). There was positive correlation between appearance satisfaction and self-esteem (*r* = 0.62, *p *< 0.001) and negative correlation between appearance satisfaction and body mass index (BMI) (*r* = − 0.28, *p *< 0.001). The higher the age of 1 year, the greater the probability of experience of cosmetic procedure (1.17 times) (OR 1.17, *p *= 0.002), and the higher the ACS score, the greater the probability of cosmetic procedure being 1.06 times (OR 1.06, *p *< 0.001). The higher the BMI score, the lower the probability of experiencing cosmetic procedure by 0.84 times (OR 0.84, *p *< 0.001).

**Conclusion:**

Age, ACS and BMI were the factors influencing the cosmetic procedure experience, and the cosmetic procedure experience led to more cosmetic procedures.

**Level of Evidence V:**

Opinions of respected authorities, based on clinical experience, descriptive studies, or reports of expert committees.

This journal requires that authors assign a level of evidence to each article. For a full description of these Evidence-Based Medicine ratings, please refer to the Table of Contents or the online Instructions to Authors www.springer.com/00266.

## Introduction

Standards of appearance vary according to age and country, but considering survey results [1] in which more than 65% of participants were willing to have surgical and non-surgical cosmetic procedures in relation to employment and marriage, it is clear that the self-image of an individual, which is evaluated to a significant extent through appearance, also affects perceptions of social roles in the world. Further, in the same survey, the portion of respondents who answered that appearance was reasonably important in life continuously increased from 45% in 1994, to 58% in 2004 and to 61% in 2014, indicating a growing proportion of Koreans for whom body image was important for living in the twenty-first century. This rising trend has become more widespread with the development of the Internet and mass media, and the role of appearance as a means for social enhancement has become more entrenched. In particular, Korea already has the highest global rate of cosmetic procedures relative to its population [2, 3]. This involves the younger generation in their 20s, particularly women, as women’s desire to be more beautiful goes beyond hair styles and fashion, increasing the likelihood of a more aggressive appearance change, that is, an acceptance of and willingness to have cosmetic procedures [4–6].

This high acceptance of cosmetic procedures affects in turn the likelihood of experiencing cosmetic procedures. A willingness to accept cosmetic procedures is influenced by the level of desire for cosmetic procedures and the degree of perceived necessity of cosmetic procedures for successful social life or self-improvement [6]. In Korean research on university students, factors such as sex, grade level, cosmetic procedure experience, and self-esteem have been found to influence the acceptance of cosmetic procedures [7].

Unreasonable and repetitive cosmetic procedures cause economic, physical, and psychological distress to the individual concerned, and can lead to serious maladies within families and society, such as divorce and suicide [8]. According to the Korea Consumer Agency [9], consultations on medical consumer injuries related to cosmetic procedures have shown a rising trend, and more than 100 cases have been advanced annually since 2013. Because undesirable side effects are increasing with the rise in cosmetic procedures, research is needed to understand the characteristics of cosmetic procedures, such as the level of acceptance of cosmetic surgery, self-esteem, and other related factors among those most interested in cosmetic procedures, namely women in their 20s.

The purpose of this study is to provide baseline data for the study of cosmetic procedures and for health policy settings, through a randomized survey of women in their 20s as those most concerned with and most likely to undergo cosmetic procedures, to understand better the present status of cosmetic procedures in Korea. The survey also aimed to better understand people’s experience and acceptance of cosmetic procedures.

## Methods

### Study Design

This is a descriptive study to identify and compare cosmetic procedure-related characteristics including cosmetic procedure experience and the level of acceptance of cosmetic procedures among women in their 20s.

### Data Collection

Data collection was conducted on panels registered with Macromill Embrain (http://www.panel.co.kr), a domestic online research company. The number of individuals involved was calculated using G*power 3.1.9.2 program software (http://www.gpower.hhu.de). A simple random sampling method was used for Korean women in their 20s nationwide, randomly extracting the e-mail accounts of the panel held by the research company, then sending an e-mail requesting participation in the survey. A total of 330 respondents completed the questionnaire, which met the minimum sample size of 275 (OR 1.5, significance level = 0.05, power = 0.90) required for binomial logistic regression analysis. Efforts were made to minimize survey error through using a system in which the survey no longer proceeded if the response time was significantly short or a response was missing. The basic sample consisted of female panel members in their 20s who consented to participate in the survey, and participants who did not consent or who withdrew from the survey were excluded from the study.

### Ethical Consideration

This study was approved by the Institutional Review Board of the affiliated institution regarding ethical considerations for the study participants, and was conducted after approval (JJNU - IRB-2017-025) concerning the research purpose, methods, explanation, agreements, and confidentiality.

### Measurement

#### General Characteristics and Cosmetic Procedure Characteristics of Participants

The general characteristics of the participants included age, body mass index (BMI), marital status, student status, and household economic situation. The cosmetic procedure characteristics of the participants included channels of side-effects information, experience of cosmetic procedures, types of cosmetic procedures experienced, and further cosmetic procedures considered and type.

#### Acceptance of Cosmetic Surgery

For determining the acceptance of cosmetic surgery (ACS), we used a tool developed by Henderson-King and Henderson-King [6]. The ACS tool consists of 15 items concerning personal and social reasons for cosmetic procedures, and the level of acceptance in considering cosmetic procedures. Each item was rated on a 7-point Likert scale (1 = completely disagree, 7 = completely agree), and a higher score indicated a higher willingness to accept cosmetic procedures. Reliability was estimated as Cronbach’s *α* = 0.88 at the time of tool development, and as Cronbach’s *α* = 0.93 in this study.

#### Appearance Satisfaction

The body cathexis scale as developed and revised by Secord and Jourard [10] was used to measure appearance satisfaction of the participants. This tool measures an individual’s satisfaction with the function and appearance of each part of their body on a 5-point Likert scale, with 1 indicating “very dissatisfied” and 5 indicating “very satisfied.” The original tool consists of 46 items, but through agreement among the researchers, 38 items were selected, excluding 8 items not relevant to this study. The score ranged from 38 to 190, and a higher score indicated a higher appearance satisfaction. Reliability was estimated as Cronbach’s *α* = 0.78 at the time of tool development, and Cronbach’s *α* = 0.94 in this study.

#### Self-Esteem

We used the self-esteem scale developed by Rosenberg [11]. This tool consists of 10 items rated on a 4-point Likert scale, with a higher score indicating a higher self-esteem. Reliability was estimated as Cronbach’s *α* = 0.92 at the time of tool development, and Cronbach’s *α* = 0.86 in this study.

#### Data Analysis

Collected data were analyzed using the SPSS 21.0/PC program. Descriptive statistics were used to determine general characteristics, cosmetic procedure characteristics, cosmetic surgery acceptance levels, appearance satisfaction, and self-esteem of the participants. A Chi-square test, *t* test, crosstabs, and ANOVA were performed to compare general characteristics, cosmetic surgery acceptance levels, and cosmetic procedure characteristics, according to the experience of cosmetic procedures, among participants. Pearson correlation coefficients were verified to identify the correlation between latent variables, and binary logistic regression was used to identify factors affecting cosmetic procedure experience.

## Results

### General Characteristics and Cosmetic Procedure Characteristics of Participants

The mean age of the participants was 25.02 years with a mean BMI of 21.37, and 293 (88.8%) were single or unmarried, while 37 (11.2%) were married. Eighty-three (25.2%) participants were students, while 247 (74.8%) were not students, and in terms of their perceived economic situation, 15 were classified as “high,” 238 as “middle,” and 77 as “low.”

In terms of cosmetic procedure characteristics, 323 (97.9%) had obtained side-effects information and 7 (2.1%) had not. Among those obtaining information, 185 obtained side-effects information through the “Internet,” 97 through “TV, newspapers, magazines,” 38 through “friends or family,” and 3 through a “doctor.” A total of 124 (37.6%) participants had undergone cosmetic procedures, and the mean age at the first cosmetic procedure was 21.81 (± 2.83). Of the 124 participants who had undergone cosmetic procedures, 95 had undergone surgical cosmetic surgery, with “eyelid cosmetic surgery” accounting for the highest proportion with 86 (90.5%) participants. There were 104 participants who had undergone non-surgical cosmetic procedures, with the most common being “botox injections” involving 59 (56.7%) participants. There were 195 participants who answered “yes” to considering further cosmetic procedures, with 171 (87.7%) considering surgical cosmetic procedures and 192 (98.5%) considering non-surgical cosmetic procedures. Of those considering surgical cosmetic procedures, those considering “eyelid cosmetic surgery” accounted for the highest proportion with 58 (29.7%) participants, and among those considering non-surgical cosmetic procedures, those considering “laser hair removal” accounted for the highest proportion with 131 (67.2%) participants. The mean values of the main measurements are as follows: ACS = 66.52 (± 16.67), appearance satisfaction = 122.21 (± 21.31) and self-esteem = 29.91 (± 4.83) (Table 1).

**Table 1:** General characteristics and cosmetic procedure characteristics of cosmetic surgery of participants (*N *= 330)

Variables	Categories	*n* (%) or *M* ± SD
Age		25.02 ± 2.64
Body mass index		21.37 ± 3.51
Marital status	Yes	37 (11.2)
	No	293 (88.8)
Student status	Yes	83 (25.2)
	No	247 (74.8)
Perceived economic situation	High	15 (4.6)
	Middle	238 (72.1)
	Low	77 (23.3)
Side-effects information	Yes	323 (97.9)
	Internet	185 (57.3)
	TV, newspapers, magazines	97 (30.0)
	Friends or family	38 (11.8)
	Doctor	3 (0.9)
	No	7 (2.1)
Experience of cosmetic	Yes	124 (37.6)
procedures	Age of first cosmetic surgery	21.81 ± 2.83 (16 ~ 29)
	Surgical cosmetic surgery^a^	95 (76.6)
	Non-surgical cosmetic procedures^a^	104 (83.9)
	No	206 (62.4)
Considering further cosmetic procedures	Yes	195 (59.1)
	Surgical cosmetic surgery^a^	171 (87.7)
	Non-surgical cosmetic procedures^a^	192 (98.5)
	No	135 (40.9)
Acceptance of cosmetic surgery		66.52 ± 16.67
Appearance satisfaction		122.21 ± 21.31
Self-esteem		29.91 ± 4.83

^a^Multiple responses

### Differences in General Characteristics and Cosmetic Procedure Characteristics According to the Experience of Cosmetic Procedures Among Participants

Among the general characteristics according to participants’ experience of cosmetic procedures, significant differences were found in mean age (*t* = 3.81, *p *< 0.001), average BMI (*t* = − 3.62, *p *< 0.001), marital status (*x*^2^ = 4.82, *p *= 0.028), student status (*x*^2^ = 10.19, *p *= 0.001), considering further cosmetic procedures (*x*^2^ = 286.93, *p *< 0.001), and ACS (*t* = 7.80, *p *< 0.001) (Table 2).

**Table 2:** Differences in general characteristics and cosmetic procedure characteristics according to the experience of cosmetic procedures among participants (*N *= 330)

Variables	Categories	Experience of cosmetic procedure		*t* or *x*^2^	*p*
		Yes	No		
		*n* (%) or *M* ± SD	*n* (%) or *M* ± SD		
Age		25.69 ± 2.26	24.62 ± 2.78	3.81	< 0.001
Body mass index		20.55 ± 2.73	21.86 ± 3.83	− 3.62	< 0.001
Marital status	Yes	20 (6.1)	17 (5.1)	4.82	0.028
	No	104 (31.5)	189 (57.3)		
Student status	Yes	19 (5.8)	64 (19.4)	10.19	0.001
	No	105 (31.8)	142 (43.0)		
Perceived economic situation	High	7 (2.1)	8 (2.4)	0.57	0.754
	Middle	88 (26.7)	150 (45.4)		
	Low	29 (8.8)	48 (14.6)		
Side-effects information^a^	Yes	120 (36.4)	203 (61.5)	0.28	0.432
	No	4 (1.2)	3 (0.9)		
Considering further cosmetic procedures^a^	Yes	0 (0)	195 (59.1)	286.93	< 0.001
	No	124 (37.6)	11 (3.3)		
Acceptance of cosmetic surgery		74.59 ± 13.14	61.67 ± 16.71	7.80	< 0.001
Appearance satisfaction		119.62 ± 19.09	12.77 ± 22.45	− 1.72	0.087
Self-esteem		30.08 ± 4.94	29.81 ± 4.77	0.50	0.617

^a^Fisher’s exact test

### ACS Differences According to General Characteristics and Cosmetic Procedure Characteristics of Participants

For ACS according to the general characteristics of participants, there was a significant difference according to their experience of cosmetic procedures (*t* = − 7.79, *p *< 0.001) and considering further cosmetic procedures (*t* = 5.05, *p *< 0.001) (Table 3).

**Table 3:** ACS differences according to general characteristics and cosmetic procedure characteristics of participants (*N *= 330)

Variables	Categories	ACS *M* ± SD	*t* or *F*	*p*
Marital status	Yes	70.14 ± 15.79	− 1.40	0.162
	No	66.07 ± 16.74		
Student	Yes	65.83 ± 14.03	− 0.44	0.662
	No	66.76 ± 17.48		
Perceived economic situation	High	61.73 ± 18.20	0.99	0.373
	Middle	67.19 ± 16.14		
	Low	66.52 ± 16.67		
Side-effects information	Yes	66.64 ± 16.57	0.89	0.376
	No	61.00 ± 21.50		
Experience of cosmetic procedures	Yes	74.59 ± 13.14	− 7.79	< 0.001
	No	61.67 ± 16.71		
Considering further cosmetic procedures	Yes	62.81 ± 16.28	5.05	< 0.001
	No	71.90 ± 15.78		

ACS = acceptance of cosmetic surgery

### Correlation Among the Main Variables

The participants’ self-esteem and BMI showed a negative correlation (*r* = − 0.13, *p *= 0.024). The appearance satisfaction of participants showed a negative correlation with BMI (*r* = − 0.28, *p *< 0.001) and ACS (*r* = − 0.18, *p *= 0.001), and a positive correlation with self-esteem (*r* = 0.62, *p *< 0.001) (Table 4).

**Table 4:** Correlation among the main variables

Variables	Age *r*(*p*)	BMI *r*(*p*)	ACS *r*(*p*)	Appearance satisfaction *r*(*p*)	Self-esteem *r*(*p*)
Age	1				
BMI	− 0.04 (.438)	1			
ACS	0.08 (.130)	− 0.01 (0.882)	1		
Appearance satisfaction	− 0.05 (.328)	− 0.28 (< 0.001)	− 0.18 (0.001)	1	
Self-esteem	0.05 (.315)	− 0.13 (0.024)	0.03 (0.647)	0.62 (< 0.001)	1

ACS = acceptance of cosmetic surgery, BMI = body mass index

### Influencing Factors on the Experience of Cosmetic Procedures

To identify the factors affecting cosmetic procedure experience, a binominal logistic regression analysis was performed including the variables showing significant differences in this study. The binomial logistic regression analysis showed that significance was 0.962 in the Hosmer and Lemeshow tests, with a significance greater than 0.05 indicating a good model fit. The independent variables were found to affect cosmetic procedure experience (*x*^2^ = 78.63, *p *< 0.001), and the explanatory power of the variables was 28.9%. Among the variables in the regression model, age, ACS, and BMI were found to be significant variables. In other words, as age increased by 1 year, the probability of having experienced cosmetic procedures increased 1.17 times; as ACS increased by 1 point, the probability of having experienced cosmetic procedures increased 1.06 times; and as BMI increased by 1 point, the probability of having experienced cosmetic procedures decreased 0.84 times (Table 5).

**Table 5:** Influencing factors on the experience of cosmetic procedures

	B	SE	Wald	*p*	OR (95% CI)
Age	0.16	0.05	9.49	0.002	1.17 (1.06–1.29)
BMI	− 0.17	0.05	13.82	< 0.001	0.84 (0.77–0.92)
ACS	0.06	0.01	34.80	< 0.001	1.06 (1.04–1.08)
Appearance satisfaction	− 0.01	0.01	2.98	0.084	0.99 (0.97–1.00)
Self-esteem	0.03	0.04	0.78	0.377	1.03 (0.96–1.11)

*OR* odds ratio (experience of cosmetic surgery: no = 0, yes = 1)

ACS = acceptance of cosmetic surgery, BMI = body mass index

## Discussion

This study investigated the experience of cosmetic procedures, perception of side effects, levels of acceptance, and the factors influencing the experience of cosmetic procedures among Korean women in their 20s.

The mean age of participants was 25.02 years. Although 97.9% of participants had obtained information on the side effects of cosmetic procedures, most had received information through the “Internet” or “TV, newspaper, magazine,” whereas obtaining such information through a “doctor” was very low at 0.9%. This rate for those obtaining side-effects information and the rate for using the “Internet” and “TV, newspaper, magazine” as channels of awareness were higher in this study than in results by Kim et al. [7]. In addition, 37.6% of participants had undergone 1 or more cosmetic procedures, and 59.0% answered “yes” to considering further cosmetic procedures. Amid increasing cases of consultation in relation to side effects involving cosmetic procedures [9], in addition to consideration of individual experiences and expectations concerning cosmetic procedures, it is necessary to find a means to increase the diffusion of side-effects information on cosmetic procedures through medical experts.

In this study, the ACS score was 66.52, which was higher than the 60.89 score found in a previous study of undergraduate students [7]. This difference was considered to arise due to differences among the participants, because students accounted for approximately a quarter of the participants in this study; the majority of participants were women fully engaged in social life through work or other commitments, and that it was likely that an internalization of social standards in respect to appearance had already been made to some extent.

Appearance satisfaction displayed a positive correlation with self-esteem and a negative correlation with BMI. This finding supports previous results showing that there was a positive correlation between BMI and body dissatisfaction [12], and that high BMI values were predictors of high body/body image dissatisfaction [13, 14]. In addition, appearance satisfaction was negatively correlated with ACS in this study. This result was also similar to previous study results [15] showing that body satisfaction was a negative influence factor on ACS. Considering these results, it could be assumed that a high BMI for women in their 20s was associated with low body image, and that low body image and low appearance satisfaction were likely to lead to a decrease in self-esteem. The social assessment of appearance affects self-esteem [16], and low self-esteem related to BMI among women in their 20s who have just entered the working arena can lead to depression [14]. As approximately three quarters of the participants in this study were not students but had just began their social working-life experience in their 20s, it is highly likely that they would have experienced appearance evaluation through work or part-time jobs, either directly or indirectly. Given awareness that levels of self-esteem according to appearance satisfaction can be directly linked to the health of women in their 20s, a comprehensive means to bring about changes in social norms and values in respect of appearance satisfaction should also be sought.

The study participants were 1.17 more times likely to have had cosmetic procedure experience as their age increased by 1 year. This could be interpreted as due to the probability of having had increasing experience of cosmetic procedures, as well as of reaching an age where it was easier to try more aggressive methods to meet appearance satisfaction and where the participants were exposed to more competition in areas such as employment. Support for these findings could also be inferred through the results of a study that found the lower the self-esteem of high school girls, the higher the desire for cosmetic procedures [17], and a study finding that body dissatisfaction among male and female adolescents increases with age [13]. As the probability of selecting cosmetic surgery increases with age, following entry into adulthood after adolescence when interest in appearance rapidly increases; it is necessary to identify and apply effective measures for improving self-esteem before adolescence.

The study participants were 1.06 more times likely to have had cosmetic surgery experience as their ACS score increased by 1 point. This finding supports the research that suggests people with a personal or indirect experience of cosmetic procedures are more likely to accept undergoing future cosmetic procedures [4, 7, 18]. Past cosmetic procedure experience leads to an increased interest in self-care after a cosmetic procedure, and it seems likely that a mindset that wishes to promote acceptance of one’s cosmetic surgery experience also plays a role in influencing acceptance of future cosmetic procedures.

The study participants were 0.84 times less likely to have had cosmetic procedure experience as their BMI score increased by 1 point. This finding supports research by Swami [19] that reported consideration for cosmetic procedures and BMI was negatively correlated, but diverges from the research findings of Henderson-King and Henderson-King [6] in which a negative correlation was found only among men. Further, as our finding contradicts another Korean study [17] that found that a desire for cosmetic procedures increased with a higher BMI, there needs to be further studies on the relationship between BMI and ACS.

Young women are more interested in dieting than any other group, even when BMI is normal [20]. In particular, many Korean women in their 20s are exposed to nutritional imbalance and health problems through attempting excessive weight loss even when they are not overweight or obese [12, 21, 22]. Considering a study [23] that showed that those who diet or use dietary supplements were more satisfied with their body shape than those who did not, there is a need to explore various relevant variables that affect the appearance satisfaction of women in their 20s. In addition, related policies and publicity are needed to promote healthy and widely shared values relating to appearance. We recommend a long-term follow-up study, involving more population groups, concerning both the willingness to undergo cosmetic procedures and undergoing them, despite a high awareness of the side effects of cosmetic procedures.

## Conclusion

This study aimed to provide relevant baseline data for measures and policies to improve the body image and the self-esteem of young women living in modern society, through comparing the cosmetic procedure experience and the cosmetic procedure characteristics of Korean women in their 20s, and understanding better what was involved. The results of our survey conducted using random sampling of 330 women in their 20s showed that appearance satisfaction had a negative correlation with ACS and BMI, a positive correlation with self-esteem, and that age, ACS, and BMI affect the cosmetic procedure experience. The limitation of this study was that it did not investigate perceptions of cosmetic procedure side effects in more detail.

As the demand for cosmetic procedures in Korea continues to increase, it is important for society generally to understand the relevant aspects of the cosmetic procedure experience and how and why choices are made regarding cosmetic procedures. Considering that the phenomenon of body image concern in society appears to be spreading beyond the boundaries of sex, we suggest a follow-up study of women and men in their 20s to clarify the relationship between sociocultural-related variables and personal characteristics that affect the cosmetic procedure experience in addition to appearance satisfaction. Furthermore, along with a cross-sectional study, we recommend a longitudinal study on appearance satisfaction, ACS, cosmetic procedure experience, appearance satisfaction following cosmetic procedures, and self-esteem, in relation to general characteristics.
